# Radiation damage to nucleoprotein complexes in macromolecular crystallography

**DOI:** 10.1107/S1600577514026289

**Published:** 2015-01-30

**Authors:** Charles Bury, Elspeth F. Garman, Helen Mary Ginn, Raimond B. G. Ravelli, Ian Carmichael, Geoff Kneale, John E. McGeehan

**Affiliations:** a Laboratory of Molecular Biophysics, Department of Biochemistry, University of Oxford, South Parks Road, Oxford OX1 3QU, UK; b Institute of Nanoscopy, Maastricht University, PO Box 616, Maastricht 6200 MD, The Netherlands; c Notre Dame Radiation Laboratory, University of Notre Dame, Notre Dame, IN 46556, USA; d Molecular Biophysics, Institute of Biomedical and Biomolecular Sciences, University of Portsmouth, King Henry 1st Street, Portsmouth PO1 2DY, UK

**Keywords:** macromolecular crystallography, radiation damage, protein–DNA complexes, specific damage

## Abstract

Significant progress has been made in macromolecular crystallography over recent years in both the understanding and mitigation of X-ray induced radiation damage when collecting diffraction data from crystalline proteins. In contrast, despite the large field that is productively engaged in the study of radiation chemistry of nucleic acids, particularly of DNA, there are currently very few X-ray crystallographic studies on radiation damage mechanisms in nucleic acids. Quantitative comparison of damage to protein and DNA crystals separately is challenging, but many of the issues are circumvented by studying pre-formed biological nucleoprotein complexes where direct comparison of each component can be made under the same controlled conditions. Here a model protein–DNA complex C.Esp1396I is employed to investigate specific damage mechanisms for protein and DNA in a biologically relevant complex over a large dose range (2.07–44.63 MGy). In order to allow a quantitative analysis of radiation damage sites from a complex series of macromolecular diffraction data, a computational method has been developed that is generally applicable to the field. Typical specific damage was observed for both the protein on particular amino acids and for the DNA on, for example, the cleavage of base-sugar N_1_—C and sugar-phosphate C—O bonds. Strikingly the DNA component was determined to be far more resistant to specific damage than the protein for the investigated dose range. At low doses the protein was observed to be susceptible to radiation damage while the DNA was far more resistant, damage only being observed at significantly higher doses.

## Abstract 2

Quantitative X-ray induced radiation damage studies employing a model protein–DNA complex revealed a striking partition of damage sites. The DNA component was observed to be far more resistant to specific damage compared with the protein.

## 1. Introduction   

Since the advent of powerful third-generation synchrotron sources, significant progress has been made in the field of X-ray crystallography regarding the analysis of X-ray induced radiation damage to proteins during both 100 K and room temperature (RT) diffraction data collection. A collection of careful systematic studies has increased awareness of the issues and has served to provide practical solutions, from optimized data-collection strategies (Zeldin *et al.*, 2013*a* ▶; Flot *et al.*, 2010 ▶) through to the addition of free-radical scavengers, to help mitigate the destructive effects [for a summary of scavenger studies see Allan *et al.* (2013 ▶)]. As we strive to solve the structures of macromolecules with ever-increasing complexity, it is also important to consider the effects of radiation damage to fundamental non-protein biological components. A wealth of radiation damage studies for nucleic acids has been provided by a strong community of radiation chemists, and mechanisms have been deduced from experiments on individual nucleotides in isolation through to irradiation of whole cells and tissues. The latter studies underpin the development of current radiotherapies in the treatment of a range of cancers, but the full mechanistic X-ray damage landscape from atoms to organisms is far from complete. The atomic resolution that can be provided by X-ray crystallography has great potential to help link the fields of radiation chemistry and radiation biology by providing an atomistic view of radiation damage to intact biological complexes, particularly those involving nucleoproteins.

Obtaining an understanding of damage to these complexes is important because DNA is rarely naked in a cell, operating instead in a dynamic environment interacting with a plethora of proteins such as the nucleosomal histones through to high-order replication, transcription, modification and repair machinery. In contrast with X-ray radiation damage investigations on isolated crystallized proteins or nucleic acids, there has been relatively little work focused on the quantitative comparison of damage observations between the two. Radiation damage solution studies of biological complexes, including the lac repressor-operator (Begusová *et al.*, 2001 ▶; Charlier *et al.*, 2002 ▶; Eon *et al.*, 2001 ▶) and estrogen response element-receptor complexes (Stisova *et al.*, 2006 ▶), suggest a significant bias towards protein damage as compared with the nucleic acid component (reviewed by Spotheim-Maurizot & Davídková, 2011 ▶). Previous studies have highlighted the importance of differential radiation induced damage and its effect on the interactions required to form nucleoprotein complexes. It has been shown that bound DNA affords a substantial protective effect in the complex between the DNA glycosylase Fpg and its abasic DNA target site (Gillard *et al.*, 2004 ▶) and that the naked protein has relatively higher sensitivity to irradiative damage. The authors note that this may be a common feature in DNA repair enzymes and may make a significant contribution to biological damage processes. Furthermore, subsequent studies by the same group on the lac-operator model system were able to identify damage to specific amino acids of the DNA binding protein component using a combination of spectroscopy and mass spectrometry (Gillard *et al.*, 2007 ▶). In the current study, we aim to build on this information by employing techniques that will allow us to investigate these radiation induced damage processes at the atomic level.

In macromolecular crystallographic investigations, direct quantitative comparison between damage to different proteins in different crystalline conditions is intrinsically challenging and this is made yet more difficult when one considers nucleic acid crystals. DNA crystals, for example, often form pseudo-continuous helical lattices that are far from representative of their solution state. On the other hand, the ability to generate crystals of protein–DNA and protein–RNA complexes provides a tractable basis to compare relative damage profiles between the two components under the same controlled experimental conditions.

X-ray radiation damage is generally recognized as the major limitation in macromolecular crystallography (MX), resulting in loss of diffraction data quality from ‘global radiation damage’, observed in reciprocal space as loss of diffraction intensity, loss of resolution, unit-cell expansion and, usually, increased mosaicity (Murray & Garman, 2002 ▶; Owen *et al.*, 2006 ▶; Ravelli & McSweeney, 2000 ▶; Burmeister, 2000 ▶; Garman, 2010 ▶). Furthermore, radiation damage can be the result of localized chemical changes to the protein structure, ‘specific radiation damage’, which at 100 K data collection temperatures is observed to follow a reproducible general trend. These effects include reduction of metallo-centres, disulfide bond breakage, acidic residue decarboxylation, hydroxyl group loss from tyrosines and methylthio group damage, all of which can compromise biological data interpretation (Weik *et al.*, 2000 ▶; Ravelli & McSweeney, 2000 ▶; Burmeister, 2000 ▶; Fioravanti *et al.*, 2007 ▶; Garman & Owen, 2006 ▶; Ravelli & Garman, 2006 ▶). Radiation damage can similarly result in specific site damage to nucleic acids such as base-sugar N_1_—C or sugar-phosphate C—O bond cleavage in DNA (Théodore *et al.*, 2006 ▶).

The major underlying cause of radiation damage is the photoabsorption in the crystal (solvent and macromolecule) of incoming X-rays. The resulting high-energy photoelectron, typically with a range of 3 to 4 µm for the incident X-ray energies used in MX (Nave & Hill, 2005 ▶; Sanishvili *et al.*, 2011 ▶) as well as associated Auger electrons, can produce a cascade of many hundreds of further ionization and excitation events (O’Neill *et al.*, 2002 ▶) releasing secondary electrons which then lose energy to the medium and gradually thermalize. Inelastic Compton scattering of incoming X-rays provides another source of ionizing electrons which are also mobile in the crystal at 100 K and help spread the damage. Direct damage refers to ionization events occurring within the protein or DNA molecule, and indirect damage is that transferred from the effects of absorption in the surrounding solvent.

There have been numerous studies devoted to understanding the radiation damage mechanisms in DNA, involving resonant attachment to either the bases or the sugar-phosphate backbone, by which secondary sub-ionization-inducing low-energy electrons (0–15 eV) inflict this damage (Michael & O’Neill, 2000 ▶; Boudaïffa *et al.*, 2000 ▶; Alizadeh *et al.*, 2014 ▶; Ptasińska & Sanche, 2007 ▶; Alizadeh & Sanche, 2014 ▶). This attachment can result in covalent bond cleavage of the DNA backbone, observed as single strand breaks (SSBs) (Boudaïffa *et al.*, 2000 ▶; Simons, 2006 ▶; Barrios *et al.*, 2002 ▶; Berdys *et al.*, 2004 ▶; Michael & O’Neill, 2000 ▶). Furthermore, other investigations have shown suspected preferential cleavage of sugar-phosphate C—O bonds over base-sugar N_1_—C bonds and other bonds present within the DNA structure (Théodore *et al.*, 2006 ▶; Ptasińska & Sanche, 2007 ▶).

Other X-ray induced DNA damage studies have demonstrated the importance of the secondary hydroxyl radicals (OH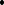) produced by water radiolysis within solvent channels throughout the crystal structure, which then can proceed to abstract H atoms from deoxyribose and bases (Michael & O’Neill, 2000 ▶; Cadet *et al.*, 1999 ▶). Alternatively hydroxyl radicals can add to unsaturated linkages within DNA bases, resulting in SSBs and modified bases and sugars (Spotheim-Maurizot & Davídková, 2011 ▶). In fact it has been estimated that two-thirds of DNA damage in dilute aqueous solution at room temperature can be attributed to ‘indirect’ secondary free radical damage from solvent around the DNA (Michael & O’Neill, 2000 ▶).

In this paper, a well characterized bacterial protein–DNA complex (C.Esp1396I/DNA) is employed to probe the effects of X-ray induced radiation damage and its quantitative distribution among the various components at the atomic level.

The C-protein C.Esp1396I is a member of a large class of helix-turn-helix proteins that act as transcriptional regulators of gene expression in both prokaryotic and eukaryotic systems. C.Esp1396I finely regulates a bacterial restriction-modification system, providing a delay in endonuclease expression *via* a concentration-dependent ‘on’ and ‘off’ switch. This type of transcriptional regulation is a cornerstone of all biological systems, and requires the recognition and intimate interaction of proteins and specific DNA target sites to form productive complexes.

The C.Esp1396I nucleoprotein tetramer complex [PDB ID: 3clc, resolution 2.8 Å (McGeehan *et al.*, 2008 ▶)] was chosen as a model due to the relatively large 35 bp dsDNA component, resulting in the number of protein and DNA atoms being of the same order (protein: 2496 non-hydrogen atoms; DNA: 1429 atoms). This allowed a statistically significant comparison between the frequencies of detected specific damage within the DNA and protein components. In addition, having four identical subunits in the complex as well as pseudo-symmetry in the DNA provides a further indicator of statistical significance.

## 2. Materials and methods   

### 2.1. Crystallization and X-ray data collection   

Expression, purification and crystallization of the native C-protein–DNA operator complex C.Esp1396I was performed as described earlier (McGeehan *et al.*, 2008 ▶). Pure C.Esp1396I protein at a concentration of 0.7 mg ml^−1^ was mixed with purified double-stranded 35 bp DNA at a 4:1 molar ratio and then crystallized using a vapour-diffusion sitting-drop method at 293 K with 100 nl nucleoprotein solution and 100 nl mother liquor drop. Crystals grew within one month in 50 m*M* 2-(*N*-morpholino)ethanesulfonic acid (MES), pH 7.5, 25% 2-methyl-2,4-pentanediol (*v*/*v*) and 40 m*M* MgCl_2_. They were cryoloop mounted and vitrified directly under a 100 K N_2_ gas stream, with no additional cryoprotectant agent being added to the mother liquor.

Data were collected at 100 K at beamline ID29, ESRF, using a wavelength of 0.932 Å (13.3 keV) and an ADSC Q315R mosaic CCD detector. A pinhole designed by R. Ravelli and F. Felisaz (EMBL Grenoble) was utilized to produce a 25 µm circular low-divergence beam at the sample. The beam size before the pinhole was 0.212 mm (vertical) × 0.279 mm (horizontal) (Gaussian profile FWHM). A crystal of C.Esp1396I (30 µm × 30 µm × 10 µm) was oriented with the beam direction parallel to the smallest crystal dimension (10 µm). The crystal–detector distance was fixed to 390 mm throughout.

Eight datasets were collected from this C.Esp1396I crystal, each consisting of 100 frames of 1° rotation, each over the same 100° wedge of the crystal, with exposure times per frame and beam attenuation being varied according to the values shown in Table 1 ▶. The unattenuated beam (100% beam transmission) used for dataset 7 was included to damage the crystal at a higher rate than in datasets 1–6, and ensure that data had been collected for a large range of absorbed X-ray doses by the last dataset.

### 2.2. Dose calculation   

In order to interpret the X-ray radiation damage data it is crucial to have an accurate estimate of the absorbed X-ray dose. Based on the image of the crystal mounted on the beamline (Fig. 2*a*), RADDOSE-3D (Zeldin *et al.*, 2013*b* ▶), which now includes a recently developed capability to handle polygonal crystals, was used to calculate the absorbed dose distributions after the first (Fig. 2*b*) and eighth (Fig. 2*c*) datasets. The intensity profile of the 25 µm-diameter beam was modelled as a Gaussian with FWHM values as detailed above. The photon flux prior to attenuation was estimated to be 5 × 10^10^ photons s^−1^ throughout the experiment, and the resulting diffraction weighted dose (DWD) values (Zeldin *et al.*, 2013*a* ▶) for each dataset are shown in Table 1 ▶. The flux value was obtained from the in-beamline ionization gauge I1 which had been cross-checked with a calibrated diode (Owen *et al.*, 2009 ▶) the day before the data collection.

The calculation of the crystal absorption coefficients in RADDOSE-3D included the water and the heavy-atom content from the crystallization conditions (the sulfur in the MES buffer and the magnesium and chlorine from the MgCl_2_). All the results on radiation progression were plotted against the resulting DWD values. A plot of the mean intensity values per whole dataset showing the radiation damage induced decay can be found in Fig. S1 of the supporting information.^1^ The dose to half mean intensity, 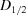, is approximately 45 MGy, in good agreement with the experimental 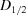 dose limit of 43 MGy (Owen *et al.*, 2006 ▶).

### 2.3. Data processing   

Each dataset was integrated using *iMOSFLM* (Leslie & Powell, 2007 ▶) and scaled using the CCP4 program *AIMLESS* (Winn *et al.*, 2011 ▶). For the first dataset, molecular replacement was then performed using the program *PHASER* (McCoy *et al.*, 2007 ▶) with the previously deposited C.Esp1396I complex structure PDB entry 3clc (McGeehan *et al.*, 2008 ▶) as the search model.

The structure obtained from the first dataset was refined, initially using rigid-body refinement in *REFMAC5* (Murshudov *et al.*, 1997 ▶), followed by repeated cycles of restrained, TLS and isotropic *B*-factor refinement. This was coupled with manual inspection and refinement in *Coot* (Emsley *et al.*, 2010 ▶), involving solvent molecule removal and addition. In *REFMAC5*, non-crystallographic symmetry (NCS) restraints were used throughout for the four protein subunits present, except in the flexible loop region corresponding to residues 43–46 (Ball *et al.*, 2009 ▶). Final rounds of TLS, restrained and isotropic *B*-factor refinement were then performed for the first dataset using *phenix.refine* in the *PHENIX* program suite (Adams *et al.*, 2010 ▶), utilizing the integrated *MolProbity4* (Chen *et al.*, 2010 ▶) structure validation within *phenix.refine* to manually treat Ramachandran, rotamer and bond angle/length outliers.

### 2.4. Automated specific damage location and large dataset collection   

To observe the real-space specific damage dynamics throughout the C.Esp1396I complex, the CCP4 program *CAD* was used to create a series of seven merged files combining the observed structure factor amplitudes for the first dataset *F*_o,1_ with each later dataset (*F*_o,*n*_, for *n* = 2,…, 8) individually, which were then all scaled using the CCP4 program *SCALEIT*. A set of seven Fourier difference maps *F*_o,*n*_ − *F*_o,1_, for *n* = 2,…, 8, were then calculated using the CCP4 program *FFT* (Ten Eyck, 1973 ▶) in order to determine differences in electron density distribution within the protein–DNA complex between the first dataset and each later dataset, which could then be visualized in *Coot* (Emsley *et al.*, 2010 ▶). Thus real-space observations of specific radiation damage progression within the C.Esp1396I crystal with respect to increasing dose could be made.

Fourier difference map peaks coincident with the protein–DNA complex indicated potential specific damage regions which were investigated on a case-by-case basis. For a given dose, points within the unit cell situated on an electron density σ-contouring level (*s*) have calculated electron densities (in electrons Å^−3^) of *s* times the standard deviation (σ) from the mean electron density of the Fourier difference map. Thus the higher the ±σ-contouring level in *Coot* at which a difference map peak remained present, the greater the electron density gain/loss from the mean map density was present at that specific point in space.

To provide a large dataset of electron density difference map peaks for each dose (seven in total), representing potential specific damage sites within the C.Esp1396I structure, the *FFT* program was utilized to output the coordinates of all difference map peaks above a given threshold in terms of gain/loss of electrons per Å^3^. Initial observations of the difference maps in *Coot* led to a chosen threshold of ±0.04 e Å^−3^ as being suitable to generate large datasets of specific damage sites for which damage was visible above background noise within the Fourier difference maps. In the following analysis this electron gain/loss threshold will be referred to as the *specific damage onset*. The *FFT* program also output the maximum σ-level threshold, *s*, for each located difference map peak, which could be converted into a spatially local maximum value for the gain/loss of electrons per Å^3^ for each peak.

The seven large datasets of difference map peaks were then processed using a custom-made script, written in the object-oriented scripting language *Python*. This was designed to filter the difference map peak datasets, to (*a*) remove positive peaks relating to electron density increases between the first and a given later dataset (to isolate sites of electron density loss resulting from X-ray radiation damage), and (*b*) remove electron density peaks further than a given threshold distance (Å) from the protein–DNA complex (specified by the user). This threshold was initially chosen to be 2 Å since filtering with threshold distances of 2.5 Å or greater was observed to include peaks which were clearly noise, and a ‘by-eye’ investigation of the remaining difference map peaks for the first two difference map datasets (for which the number of filtered peaks was not too high) led to the observation that all the difference map peaks corresponding to clear specific damage were below the 2 Å threshold in these datasets.

Furthermore, with a detection radius of 2 Å around each atom, electron density changes between atoms spaced at distances of up to 4 Å apart could be detected (2 × the radius). In the case of covalently bonded atoms, carboxyl acids have C—O bond lengths of ∼1.36 Å, with other C—O bond lengths being ∼1.43 Å, the C—S bond length has been reported to be ∼1.82 Å, the C—N bond length ∼1.47 Å, and the C—C bond length ∼1.54 Å (Engh & Huber, 1991 ▶); additionally van der Waals distances for interatomic contacts have been reported to typically reside in the range ∼2 Å (for H⋯H) to ∼3.2 Å (for CH_2_⋯CH_2_) (Ramachandran & Sasisekharan, 1968 ▶). Hence the chosen threshold should be sufficient to cover both atomic van der Waals radii and roughly one covalent bond length of three-dimensional space around each atom of the protein–DNA complex, to account for potential non-uniform electron distributions around each atom present. Additionally, if the detection threshold is set to approximately the atom–atom covalent bond lengths for the complex, there is a level of symmetry, since for every atom in the complex the threshold covers the atom itself and approximately the covalent bond length (or lengths if the atom is connected to multiple others) of the bond to its adjacent atom.

The command line operated script allowed a more efficient systematic sweep through the remaining difference map peaks (alongside manual *Coot* inspection) to facilitate the quick removal of those which were not suspected to be specific damage to the molecules in the C.Esp1396I crystal. For each accepted difference map peak, the closest protein–DNA complex atom was calculated, and the spatially local maximum electron loss per Å^3^ (as discussed above) for that peak was assigned to this selected atom.

**Figure 1:**
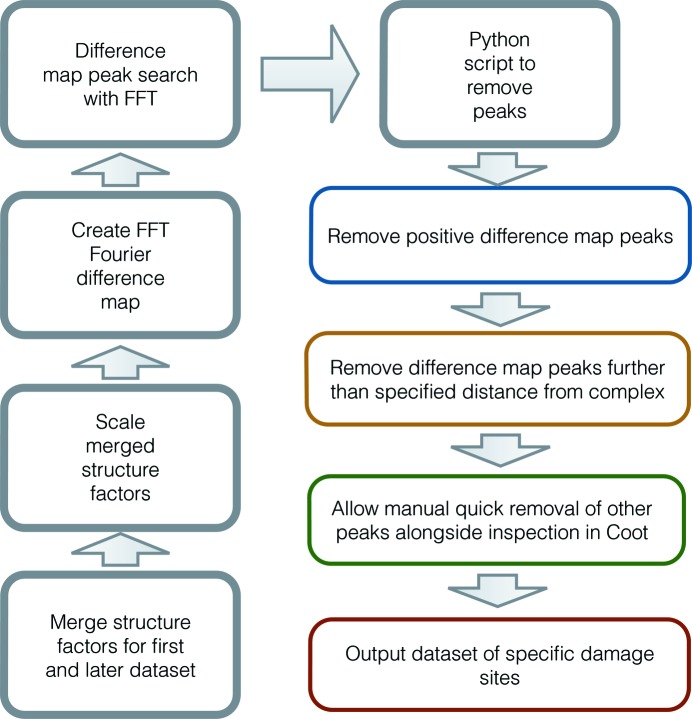
Flow chart for the difference map peak reduction process by systematic analysis, for a given later dataset *n* ∈ {2,…, 8}. Steps performed in the *Python* scripts developed for this work are coloured blue/yellow/green/red.

Thus specific damage site datasets were constructed for each Fourier difference map *F*_o,*n*_ − *F*_o,1_, for *n* = 2,…, 8, and further processed, involving the deletion of suspected specific damage sites that were detected in an earlier Fourier difference map, but then not detected in all subsequent maps. This was designed to reduce the inclusion of difference map peaks corresponding to noise, which falsely appear to represent specific radiation damage in the Fourier difference maps. For each remaining site of specific damage, the dose-dependent dynamics could then be investigated. Fig. 1 ▶ provides a flow chart representation of this analysis, in which detected *FFT*-output difference map peaks are filtered, with only suspected specific damage sites remaining.

For a given dataset *n*, the magnitude of the observed structure factor *F*_o,*n*_(000) was not known since it is equal to the total number of electrons in the unit cell, which could in principle be estimated from the amino acid composition and the m*M* buffer concentration. However, due to the uncertainty in the true solvent concentration within the crystal solvent channels, such a calculation would carry an unknown systematic error. The FFT-output electron density at real-space crystal points is given on an arbitrary fixed scale (*i.e.* multiplied by some constant scale factor *k*). For crystals differing in protein, DNA or unit-cell solvent composition, this scale factor would be different (Lang *et al.*, 2014 ▶) but, since here we are comparing structures all derived from the same crystal, the electron content of the unit is the same and thus the value of *k* is constant across the dose series and can thus be neglected in this study. Each electron density difference map peak σ-level threshold was converted into a loss/gain of electrons per Å^3^ between the first and each later dataset *via*

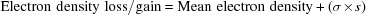
where σ is the standard deviation from the mean electron density for the difference map, and *s* is the number of standard deviations of a Fourier difference map peak from the mean electron density. Hence, for each detected difference map peak, a local electron density maximum loss/gain value was determined. These difference peaks were then illustrated using *PyMol* (Schrödinger, LLC) for structural representation and *CCP4mg* (McNicholas *et al.*, 2011 ▶) for difference maps showing specific damage examples.

## 3. Results   

### 3.1. C.Esp1396I protein–DNA complex crystallography   

**Figure 2:**
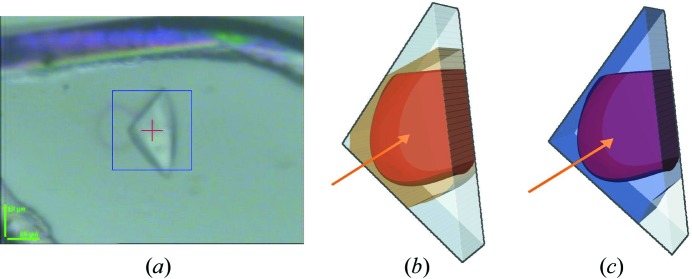
(*a*) The C.Esp1396I crystal within the rayon fibre loop during data collection. Crystal dimensions were *x* = 30 µm and *y* = 30 µm. The crystal is positioned in the loop such that dimension *z* = 10 µm is directly into the page. The blue box shown around the crystal is 50 µm × 50 µm. A faint purple ring, formed by the production of solvated electrons, can be seen to the left of the crystal. This was created from a test shot of the X-ray beam prior to crystal centring and confirms the circular 25 µm beam profile generated from the pinhole. (*b*, *c*) RADDOSE-3D (Zeldin *et al.*, 2013*b* ▶) calculation of dose distributions in the triangular C.Esp1396I crystal after (*b*) the first dataset and (*c*) the eighth dataset. In (*b*) the dose isosurfaces represent 0.16 MGy (white), 3.3 MGy (brown) and 4.2 MGy (orange) and in (*c*) 0.16 MGy (white), 20 MGy (blue) and 47.5 MGy (dark red). The direction of the X-ray beam is indicated with an arrow and the crystal was rotated about a horizontal axis.

The triangular-shaped crystal used for data collection (Fig. 2*a* ▶) was found to belong to space group *P*6_5_ with unit-cell dimensions as detailed in Table 2 ▶. In agreement with the original structure, each asymmetric unit contained one tetrameric protein–DNA complex and had a solvent content of 68.7%. The final model for the first dataset was refined to a resolution of 2.8 Å.

To investigate the distribution of specific damage throughout the C.Esp1396I protein–DNA structure with respect to increasing radiation dose, eight successive datasets on the same C.Esp1396I crystal were collected, each exposing the same 100° wedge of the crystal to X-ray radiation. For the later datasets (2 to 8), molecular replacement was performed with *PHASER* using the refined first dataset final model as the search model. The later datasets were refined only using isotropic *B*-factor refinement in *phenix.refine* and were similarly refined to 2.8 Å. Final statistics for model refinements are given in Table 2 ▶.

**Figure 3:**
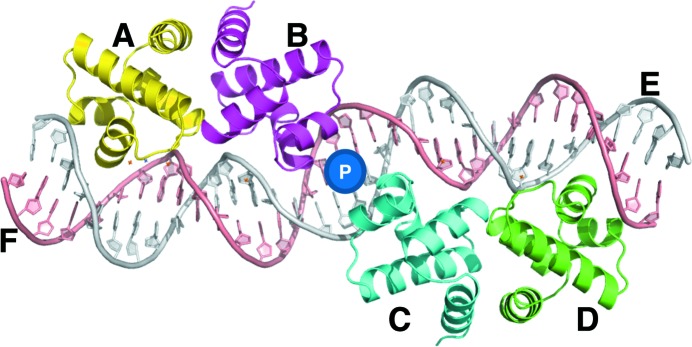
Visual representation of the C.Esp1396I complex rendered using *PyMOL*. Protein (*A*–*D*) and DNA (*E*, *F*) chains are labelled, and the 180° near-NCS symmetry axis is shown by marker *P*.

The C-protein–DNA operator complex consists of two ‘controller’ C-protein dimers (chains *A* and *B*, chains *C* and *D*) bound to a 35 bp DNA operator sequence (chains *E* and *F*) (Fig. 3 ▶) (McGeehan *et al.*, 2008 ▶). There is a pseudo-dyad axis between bp 17 and 18 of the dsDNA such that chain *A* rotates ∼180° around this axis onto chain *D* (and chain *B* onto chain *C*). Non-symmetrical binding between protein chains *B* and *C* at the dimer–dimer interface, and a pseudo-symmetrical DNA sequence, prevent true NCS in the complex. The dsDNA is distorted in the complex, due to binding of protein dimers to each operator site, resulting in minor groove compression (leading to a 50° bend at each binding site) and large major groove expansion at the DNA sequence centre (McGeehan *et al.*, 2008 ▶).

### 3.2. Specific damage observations   

**Figure 4:**
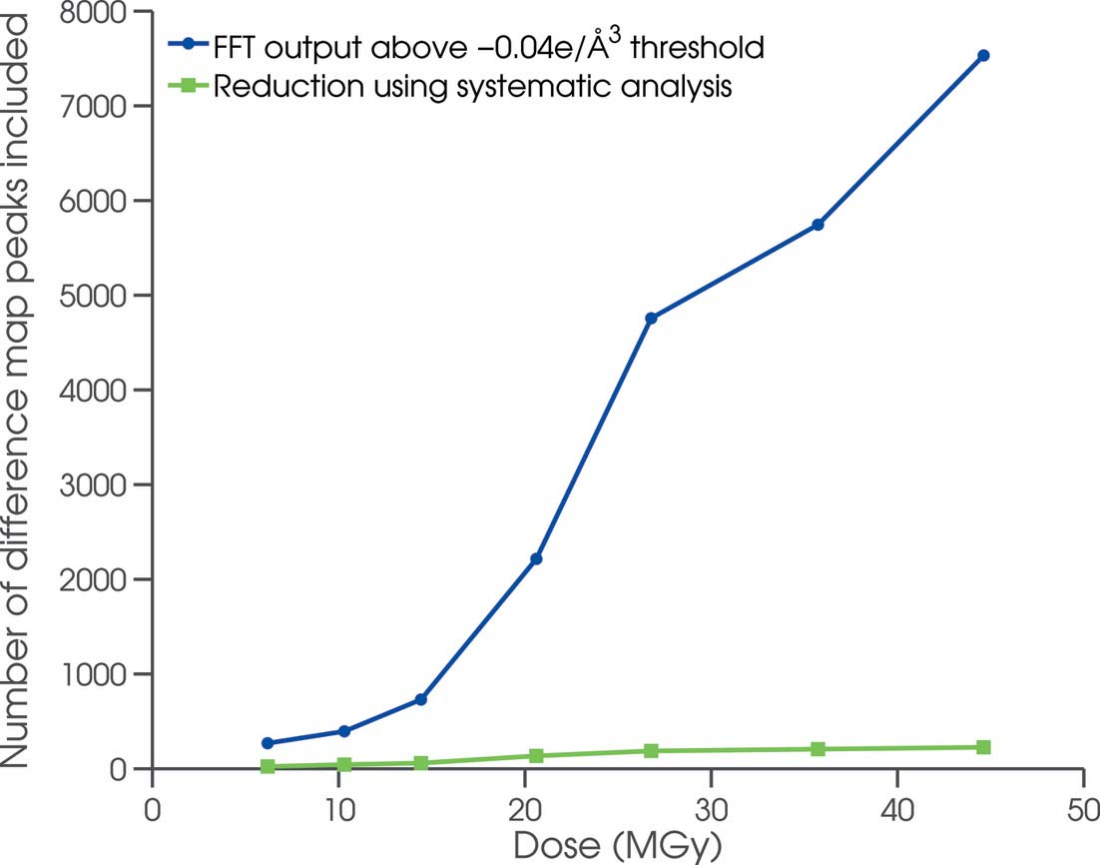
Reduction in the number of detected difference map peaks corresponding to potential specific damage obtained following a systematic analysis using the custom-made script for each dose (MGy).

**Figure 5:**
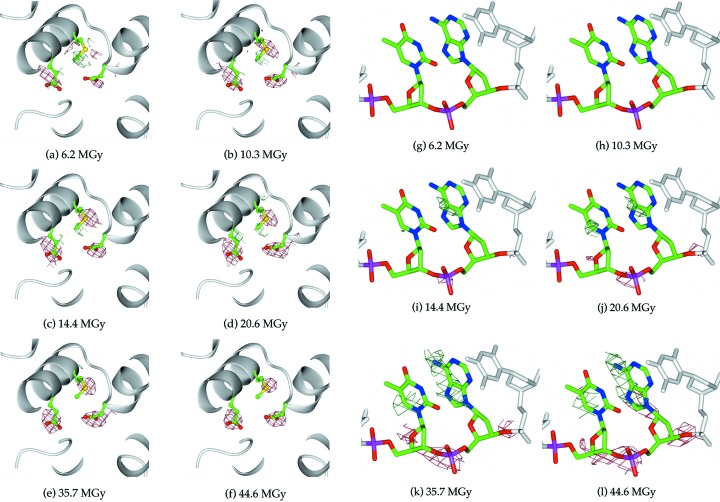
Protein and DNA damage sites in C.Esp1396I. (*a*)–(*f*) Visual representation of specific damage within protein chain *D* to Glu54, Met57 and Asp64 (green, left to right), displaying Fourier difference maps *F*_o,*n*_ − *F*_o,1_, *n* = 2,…, 7, over six increasing doses. Fourier maps are contoured at ±3.0σ in green/red. (*g*)–(*l*) Visual representation of specific damage within DNA chain *F* (with 5′ to 3′ end from left to right in each image) to T24 and A25 (green, left to right), displaying Fourier difference maps *F*_o,*n*_ − *F*_o,1_, *n* = 5,…, 8, over six increasing doses. All Fourier maps are contoured at ±3.0σ in green/red.

The automated scripts provided a means to successfully filter difference-map noise peaks from the system under investigation (Fig. 4 ▶) but they are also generally applicable for the analysis of other radiation damage datasets. With increasing radiation dose there is an associated increase in the background noise present in the Fourier difference maps due to greater dose-related disorder and non-uniformity in the configuration of each unit cell, which results in the locations of true specific site damage being obscured in high dose datasets. Filtering allowed significant reduction in the level of included noise. Indeed, Fig. 4 ▶ shows that at a dose of 45 MGy the number of difference peaks was reduced to around 1% of the original 8000 observed. Without filtering, the subsequent quantitative analysis of site-specific damage would not have been achievable over the large dose range considered here. A selection of results from this analysis is illustrated as difference maps over two regions of the complex: amino acid residues remote from the DNA binding interface on helices 4 and 5, and two nucleotides at the highly compressed TATA site between palindromic recognition sequences on the DNA. Specific damage was observed to develop throughout the C.Esp1396I complex with respect to radiation dose for both protein [Figs. 5(*a*)–5(*f*)] and DNA [Figs. 5(*g*)–5(*l*) ▶].

In terms of protein, Figs. 5(*a*)–5(*f*) reveal the specific damage dynamics of Glu54, Met57 and Asp64 in chain *D* (left to right) with respect to absorbed dose. Clear loss of electron density localized around the carboxyl groups, due to decarboxylation, is shown for Asp64 and Glu54, and the rate of carboxyl electron density loss with dose appears marginally greater for Glu54 than for Asp64. Figs. 5(*a*)–5(*f*) also show specific damage to Met57, where electron density loss and side chain disorder can be observed localized on the methylthio group over increasing doses. Although generally fewer difference peaks are observed on the DNA, there are several locations of specific damage on it too [Figs. 5(*g*)–5(*l*) ▶]. The possible sugar-phosphate C—O bond cleavage between the T24 and A25 nucleotides of DNA chain *F* would generate a single-strand break with significant biological consequences. Additionally, at higher doses [Figs. 5(*k*) and 5(*l*) ▶] positive electron density build-up is observed in close proximity to the T24 and A25 bases.

### 3.3. Chemical and topological distribution of specific damage   

**Figure 6:**
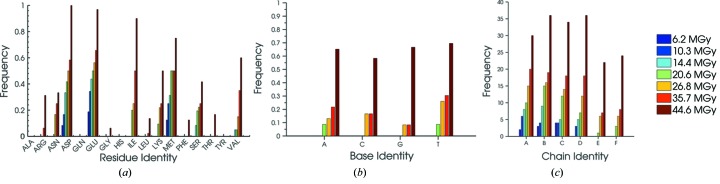
(*a*) Normalized frequency of detected specific damage against protein residue type (normalized to the frequency of occurrence of that residue in the structure) for each dose (MGy). Protein chains *A* to *D* are treated together. (*b*) Normalized frequency of detected specific damage for each DNA base type (normalized to the frequency of occurrence of that base in the structure), for each dose (MGy). DNA chains *A* to *D* are treated together. (*c*) Detected specific damage frequency for each C.Esp1396I chain (protein: *A*–*D*, DNA: *E*–*F*) for each dose.

These data allow us to investigate the location, frequency and severity of specific damage sites on a range of scales from individual chains down to residues, nucleotides and specific atoms. Fig. 6 ▶ details the distribution of detected specific damage throughout the overall C.Esp1396I complex components for each absorbed dose. For each residue type, the damage frequency is heavily dependent on the overall number of that residue present within the four protein monomers. Thus the values have been normalized by the frequency of occurrence of that particular residue in the structure (Fig. 6*a* ▶). Residues such as Asp, Glu, Met and Ser can be seen to accumulate significant damage (loss of electron density around the Ser side chain –OH) even at the lowest doses. At higher doses, damage is observed on Arg and Asn (electron density loss/disorder to the Arg/Asn main chain carboxyl group associated oxygen), Ile (partial loss of density around the side chain Cδ) and Lys (potential damage or disorder to the lysyl side chain), whilst the remaining amino acids in the protein have minimal specific damage even at very high dose. Note that C.Esp1396I has no Cys, Pro or Trp residues. There is significant heterogeneity in the rate of damage accumulation with increasing dose for each residue type. For example, by observing the step increases in damage frequency with increasing dose for each residue type, there is a clear difference in the peak detection rate between glutamate and aspartate decarboxylation for doses in the range 6.2 MGy to 35.7 MGy.

The distribution in DNA specific damage between the four nucleotide types is also shown and it is immediately apparent upon comparison with the protein data [Figs. 6(*a*) and 6(*b*) ▶] that the *specific damage onset* is at significantly higher doses for DNA than for protein within the complex, and that specific damage is more evenly distributed between the four base types than amongst the protein residues.

**Figure 7:**
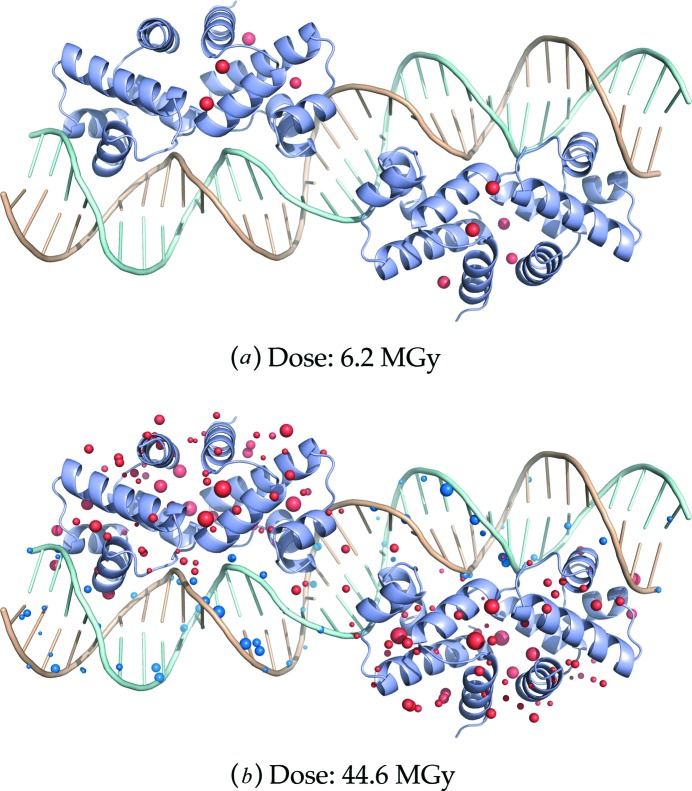
Representation of specific damage distribution throughout the C.Esp1396I complex for structures derived from the (*a*) first and (*b*) last dataset. Specific damage sites are represented as spheres, with radii proportional to electron density loss (electrons per Å^3^). Spheres closer/further than 2 Å to/from the DNA strands are coloured blue/red. Similar representations of the structures derived from the five datasets suffering intermediate doses can be found in the supporting information (Fig. S2).

Overall, there is a clear differential distribution in the dose-dependent intensities of specific damage between different protein residue types and also between the DNA and the protein. A comparison of the four protein chains *A*–*D* with the DNA chains *E* and *F* shows initial damage accumulation at >6.2 MGy *versus* >20.6 MGy, respectively (Fig. 6*c* ▶). In addition to the later onset of specific damage to the DNA chains, the frequency of detected damage peaks in them is generally lower. This observation is made more striking when these data are compiled as a visual representation of the locations of specific damage sites (above the *specific damage onset*) across the protein–DNA complex with respect to accumulating dose (Fig. 7 ▶). It is seen that, with increasing dose, (*a*) the damage site frequency increases, and (*b*) the average electron density loss magnitude increases, as expected. Furthermore, it is clearly apparent that lower-dose (Fig. 7*a* ▶ and supporting Fig. S2) damage sites are localized on the protein (predominantly chains *B* and *C*), and that even at the highest dose (Fig. 7*b* ▶), when damage sites are more homogeneously distributed throughout the protein monomers, there are still significantly fewer damage sites detected within the dsDNA component, suggesting slower time-scale damage dynamics and the existence of different damage mechanisms for the DNA.

### 3.4. Mean isotropic *B*-factor analysis   

**Figure 8:**
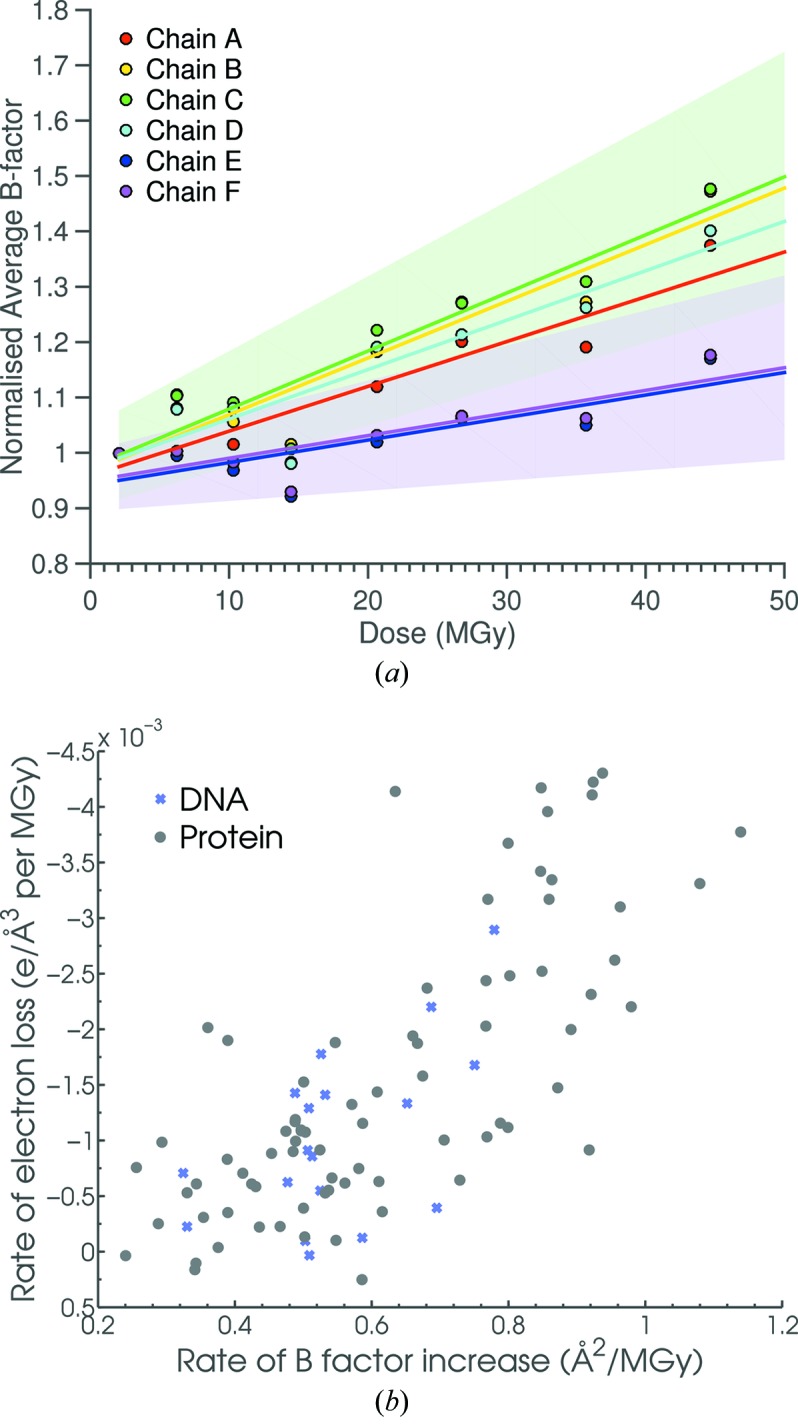
(*a*) Mean isotropic *B*-factor (Å^2^) for each chain of the C.Esp1396I complex (*A* to *D* for protein monomers, *E* and *F* for DNA single strands) against dose (MGy), normalized to the *B*-factor of the first dataset for each chain, respectively. Data points are linearly fitted for each chain. Green and purple shaded areas denote 90% confidence intervals on the intercepts and slopes for chains *C* and *F*, respectively (other chain confidence intervals are not shown in the interests of clarity). (*b*) Correlation of the average rate of isotropic *B*-factor increase (Å^2^ MGy^−1^) with the average rate of electron density loss (electrons Å^−3^ MGy^−1^). Grey/blue scatter points indicate protein/DNA specific damage sites, respectively.

Fig. 8 ▶ shows an analysis of the ‘normalized’ average *B*-factor for each chain against dose, where for each individual protein–DNA chain the ‘normalized’ *B*-factor is defined as the *B*-factor at a given dose divided by the *B*-factor at the lowest dose investigated; consequently the average *B*-factor for each chain at the lowest dose plotted is set to 1. Linear fitting for each nucleoprotein chain gave a larger rate of normalized average *B*-factor increase for each of the protein monomers than for the DNA strands (0.0081 and 0.0102 Å^2^ MGy^−1^ for protein chains *A* and *B*, respectively, and 0.0041 Å^2^ MGy^−1^ for DNA strands *E* and *F*). Furthermore, close correspondence is observed in the average isotropic *B*-factor dose-dynamics between protein chains *A* and *D* (0.0081 and 0.0089 Å^2^ MGy^−1^, respectively), protein chains *B* and *C* (0.0102 and 0.0105 Å^2^ MGy^−1^, respectively) and DNA strands *E* and *F* (both 0.0041 Å^2^ MGy^−1^), indicating the rotational near-NCS around the DNA 35 bp sequence centre.

### 3.5. Specific damage dose-dynamics   

**Figure 9:**
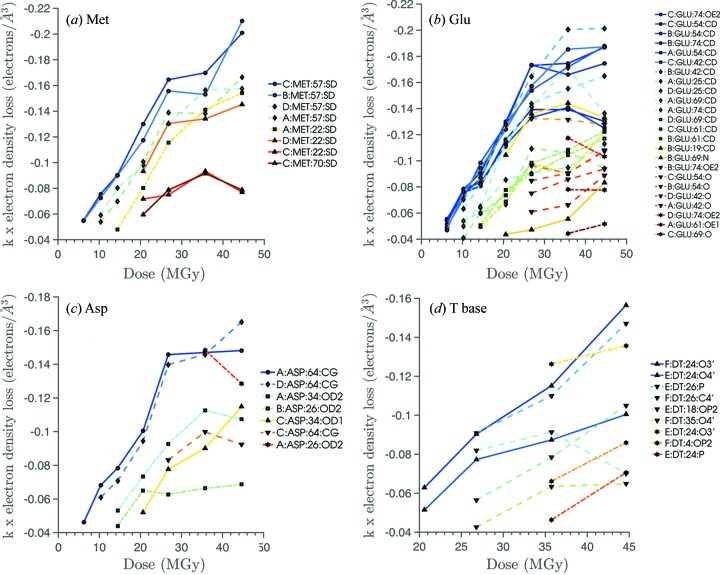
Electron density loss per Å^3^ (scaled by constant factor *k*; see §2.4) against accumulated dose (MGy) for (*a*) methylthio group damage for methionine residues, decarboxylation of (*b*) glutamate and (*c*) aspartate groups, (*d*) DNA base T damage including base-sugar N_1_—C bond and sugar-phosphate C—O bond damage.

Analysis of the electron loss per Å^3^ with respect to accumulated dose was performed for different residue types. For each subplot shown in Fig. 9 ▶, the legends detail the nearest atom of the protein–DNA complex to which specific damage has been assigned.

It is evident that clear differential specific damage rates are present not only between different residue types but also within a given residue type. For example, in Fig. 9(*a*) ▶ there is variation in the electron density loss rate with dose for the Met57 methylthio group, with the specific damage onset at lower doses for chains *B* and *C* than for the corresponding residue in chains *A* and *D*. Furthermore the dynamics of electron density loss for Met57 are qualitatively similar for chains *B* and *C*, and also for chains *A* and *D* reflecting the near non-crystallographic symmetry within the complex.

Comparing Fig. 9(*d*) with Figs. 9(*a*)–9(*c*) ▶ provides a quantitative example of the greater specific damage resistance of DNA than protein in the complex, since the specific damage onset for DNA base T is detected at significantly larger dose values (≥20 MGy) than is the specific damage for the methionine, glutamate and aspartate residue case studies which are already damaged in the first difference maps (6.2 MGy).

Analysing the average gradient for each detected damage site for electron density loss against dose (Fig. 9 ▶) and also similarly produced *B*-factor change against dose plots (not shown), the expected correlation was found between the rate of *B*-factor increase and the rate of electron density loss with respect to dose (Fig. 8*b* ▶). There appeared to be an underlying approximately linear relationship between the two metrics, reinforcing the fact that the *B*-factor increase rate with respect to absorbed dose would serve as a suitable substitute measure to monitor the heterogeneity in specific damage dynamics throughout the protein–DNA complex.

In Fig. 8(*b*) ▶  both the DNA and protein damage sites appear to follow an approximately linear trend between the average rate of *B*-factor increase and the average rate of electron density loss. However, most DNA damage sites are observed to have both low average *B*-factor increase and electron density loss rates, providing additional evidence of differential specific site dose-dynamics between the protein monomers and DNA strands.

A comparison of the heterogeneous spatial distribution in both protein residue average *B*-factor rates and protein electron density loss rates with the far more homogeneous clustering of DNA damage sites shown in Fig. 8(*b*) ▶ emphasizes that the dose-dependent dynamics of specific damage is far more uniform for DNA than for protein residues in C.Esp1396I.

## 4. Discussion   

By using an innovative highly streamlined and automated pipeline for the identification of X-ray induced structural damage patterns, we have established the existence of differential specific damage rates between the protein and DNA components of a model complex C.Esp1396I with respect to dose at 100 K. Whereas other work has studied specific protein and DNA damage in isolation (Spotheim-Maurizot & Davídková, 2011 ▶; McGeehan *et al.*, 2007 ▶; Simons, 2006 ▶; Weik *et al.*, 2000 ▶; Ravelli & McSweeney, 2000 ▶; Burmeister, 2000 ▶; Cadet *et al.*, 1999 ▶), this work investigated a large dataset of specific damage sites within a protein–DNA complex in order to produce statistically significant observations on specific damage dynamics.

The modes of action for specific damage to the protein components follow similar patterns to those documented in other studies such as decarboxylation of acidic residues and localized disruption of sulfur-containing residues, the chemistries of some of which are relatively well understood (Burmeister, 2000 ▶). We note, however, that many of the mechanisms referenced in that work were deduced from experiments carried out under quite different circumstances from those used here. For example, most radiation chemical investigations have been pursued in dilute aqueous solution. In this environment the ionizing radiation is primarily deposited in the solvent, namely water, and the holes, H_2_O^+^, rapidly deprotonate to form hydroxyl radicals, while the released electrons cause many further excitations and ionizations in the surrounding medium in the course of thermalization and solvation (Spinks & Woods, 1990 ▶). It is also important to recall that many of these aqueous radiation chemistry experiments were carried out at room temperature where both solvated electrons and hydroxyl radicals are diffusively mobile. At 100 K thermalizing electrons can still tunnel freely; however, the range explored by hydroxyl radicals may be much curtailed. The fate of ionization events directly impacting the protein would presumably be much less influenced by temperature.

An interesting example of the complexities involved might be found in the specific damage to methionine residues. In dilute aqueous solution, hydroxyl radical attack can proceed *via* 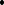
OH addition, loss of hydroxide (coupled to a water proton), forming a radical cation which subsequently deprotonates at an adjacent carbon to give a relatively persistent neutral carbon-centred radical. One possible decay channel for this species involves so-called β-cleavage, eliminating the terminal methyl, which is driven by the formation of a stabilizing CS double bond (Wisniowski *et al.*, 2002 ▶). If the medium is acidic, atomic hydrogen radicals (H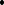), formed by the rapid neutralization reaction of the aqueous electron with ambient protons, will also be active and can, for example, also displace the terminal methyl group in an S_H_2 process (Wisniowski *et al.*, 2004 ▶). It might be expected that deprotonation at a neighbouring carbon and subsequent β-cleavage would also follow a direct hit on the methionine sulfur either from the incoming X-ray or from a released photoelectron from a nearby site.

Note that the most radiation damage susceptible linkages in proteins, namely disulfide bonds, are not present in the particular macromolecule studied here, and indeed not often present in DNA binding proteins found in the reductive intracellular environment.

In the present study, specific damage was also observed in the nucleic acid component, including evidence of a potential single-strand break (SSB) in the DNA. The location of this SSB is interesting as it correlates with a region of the DNA that is both AT-rich and under significant strain as a consequence of large-scale deformation due to protein binding. It is possible that such strained geometries enhance the radiation damage effects in DNA. This observation merits further investigation since it would have major biological consequences, as the wrapping of eukaryotic DNA around histones to form nucleosomes in part relies on the distortion of DNA around AT-rich sites such as this. The DNA radiation damage observed here could well result from previously suggested mechanisms such as low-energy secondary electron attachment to bases [a suggested precursor to SSBs (Simons, 2006 ▶)] or base modification by bonding close proximity solvent free radicals (for example, hydroxyl radical binding to carbon 6 in thymine 24) (Cadet *et al.*, 1999 ▶). However, once again it is worth noting the limited mobility of hydroxyl radicals at low temperatures, suggesting that only those formed in the immediate vicinity of the base could participate. Electrons, on the other hand, will maintain considerable mobility at 100 K.

It is again important to note that the low-energy electron damage to DNA components reported by Sanche and co-workers (Boudaïffa *et al.*, 2000 ▶; Huels *et al.*, 2003 ▶) was initially observed from DNA and components isolated on surfaces under ultrahigh-vacuum conditions. While mechanisms acting in the present protein/DNA crystalline environment might not be identical to those postulated in that investigation, recent work on low-energy electron DNA interactions is moving closer to much more relevant conditions (Alizadeh & Sanche, 2014 ▶).

We have also demonstrated that the normalized mean *B*-factor change of a particular chain gives a measure of the real-space averaged disorder present per protein–DNA chain between copies of the C.Esp1396I complex in different unit cells of the crystal. This metric indicates that, on average, the protein components become relatively more disordered with dose than does the DNA, suggesting that the protein is damaged by X-ray radiation at a faster rate than is DNA. Since any global radiation damage effects present would affect the protein and DNA components to the same extent, this could provide a suitable measure to compare specific damage susceptibility between protein and DNA. Our methodology is further validated by the identification of similar damage at palindromically equivalent sites in both protein and DNA components for this particular complex.

Further studies on a range of protein–DNA and protein–RNA complexes would allow these metrics to be tested rigorously and would reveal if our observations provide some general rules for X-ray radiation damage to biological nucleoprotein complexes. To aid this goal the custom-made scripts which allow efficient specific damage searching and enable consistent noise peak filtering could be utilized with Fourier difference maps generated from other systems. These investigations are only tractable through the use of a robust semi-automated pipeline such as the one developed here.

We can speculate that the molecule that holds our genetic blueprint has evolved to be more radiation-resistant than other cellular components, and this may not be so surprising since sacrificial proteins can be more easily replaced than lost genes. Studies on a wide range of nucleoprotein complexes utilizing these partially automated methods should provide further insight into these intriguing observations.

## Supplementary Material

1: An extension of Figure 7 representing specific damage distribution throughout the C.Esp1396I complex for structures derived from all datasets across the full dose range 6.2 MGy to 44.6 MGy. 2: Mean intensity decay for the 8 C.Esp1396I datasets.. DOI: 10.1107/S1600577514026289/xh5044sup1.pdf

PDB reference: 4x4b

PDB reference: 4x4c

PDB reference: 4x4d

PDB reference: 4x4e

PDB reference: 4x4f

PDB reference: 4x4g

PDB reference: 4x4h

PDB reference: 4x4i

## Figures and Tables

**Table 1:** Data collection details for each data set; all data sets were collected with a = 1 per image

Dataset	Exposure time per frame (s)	Transmission (%)	Cumulative dose (DWD) (MGy)
1	1	30	2.07
2	1	30	6.19
3	1	30	10.31
4	1	30	14.43
5	2	30	20.62
6	1	30	26.78
7	1	100	35.73
8	1	30	44.63

**Table 2:** Data processing and refinement statistics Values in parentheses are for the highest-resolution shells. The *B*-factor is estimated from the Wilson plot. For observed *F*_obs_ and calculated *F*_calc_ structure factors, *R*_work_ = |*F*_abs_ *F*_calc_|/*F*_obs_ and *R*_free_ is the *R*_work_ formula calculated from a small (5%) test set of randomly selected reflections, output by *phenix.refine* (Adams *et al.*). Unit-cell angles in *P*6_5_ are , and and 90, 90, 120, respectively. The resolution range is 69.52.8 with 2.952.8 for the outer shell for all datasets. A plot of the mean intensity values showing the radiation damage induced decay can be found in Fig. S1 of the supporting information.

Dataset	1	2	3	4	5	6	7	8
	4x4b	4x4c	4x4d	4x4e	4x4f	4x4g	4x4h	4x4i
Data processing								
Cell dimensions								
*a* = *b* ()	104.29	104.38	104.33	104.43	104.45	104.45	104.33	104.43
*c* ()	139.18	139.26	139.20	139.21	139.19	139.09	139.01	139.08
No. of observations	128219	129102	128743	128973	128476	129426	126479	126715
Unique reflections	21172	21231	21198	21250	21207	21246	21052	21244
Multiplicity	6.1 (6.2)	6.1 (6.2)	6.1 (6.2)	6.1 (6.2)	6.1 (6.2)	6.1 (6.2)	6.0 (6.1)	6.0 (6.1)
Completeness (%)	99.9	99.9	99.9	99.9	99.7	99.9	99.4	99.9
*R*_merge_	0.05 (0.30)	0.05 (0.33)	0.05 (0.41)	0.05 (0.47)	0.05 (0.39)	0.06 (0.82)	0.05 (0.63)	0.08 (1.92)
*I*_*n*_/*I*_1_	1.000	0.988	0.835	0.842	0.729	0.661	0.589	0.514

Refinement								
*R*_work_	0.2045	0.2338	0.2346	0.2395	0.2366	0.2336	0.2459	0.2639
*R*_free_	0.2616	0.2794	0.2824	0.2848	0.2811	0.2747	0.2929	0.2956
Mean *B*-factor (^2^)	62.08	65.23	62.93	59.59	66.42	72.59	71.96	81.63
No. non-H atoms								
Protein	2496	2496	2496	2496	2496	2496	2496	2496
DNA	1429	1429	1429	1429	1429	1429	1429	1429
Solvent	5	0	0	0	0	0	0	0
RMSD bond length ()	0.011	0.011	0.011	0.011	0.011	0.011	0.011	0.011
RMSD bond angle ()	1.338	1.338	1.338	1.338	1.338	1.338	1.338	1.338
Ramachandran								
Favoured	93.0	93.0	93.0	93.0	93.0	93.0	93.0	93.0
Outliers	6.7	6.7	6.7	6.7	6.7	6.7	6.7	6.7
Allowed	0.3	0.3	0.3	0.3	0.3	0.3	0.3	0.3

## Footnotes

1 Supporting information for this paper is available from the IUCr electronic archives (Reference: XH5044).
