# Supplementary material for: Radiation damage to nucleoprotein complexes in macromolecular crystallography
Source: J Synchrotron Radiat. 2015 Jan 30;22(Pt 2):213–24. doi: 10.1107/S1600577514026289 (PMC4344358; doi:10.1107/S1600577514026289)
Supplement: Supplementary file 1 [file s-22-00213-sup1.pdf]

JOURNAL OF SYNCHROTRON RADIATION

Volume 22 (2015)

Supporting information for article:

Radiation damage to nucleoprotein complexes in macromolecular crystallography

Charles Bury, Elspeth F. Garman, Helen Mary Ginn, Raimond B. G. Ravelli, Ian Carmichael, Geoff Kneale and John E. McGeehan

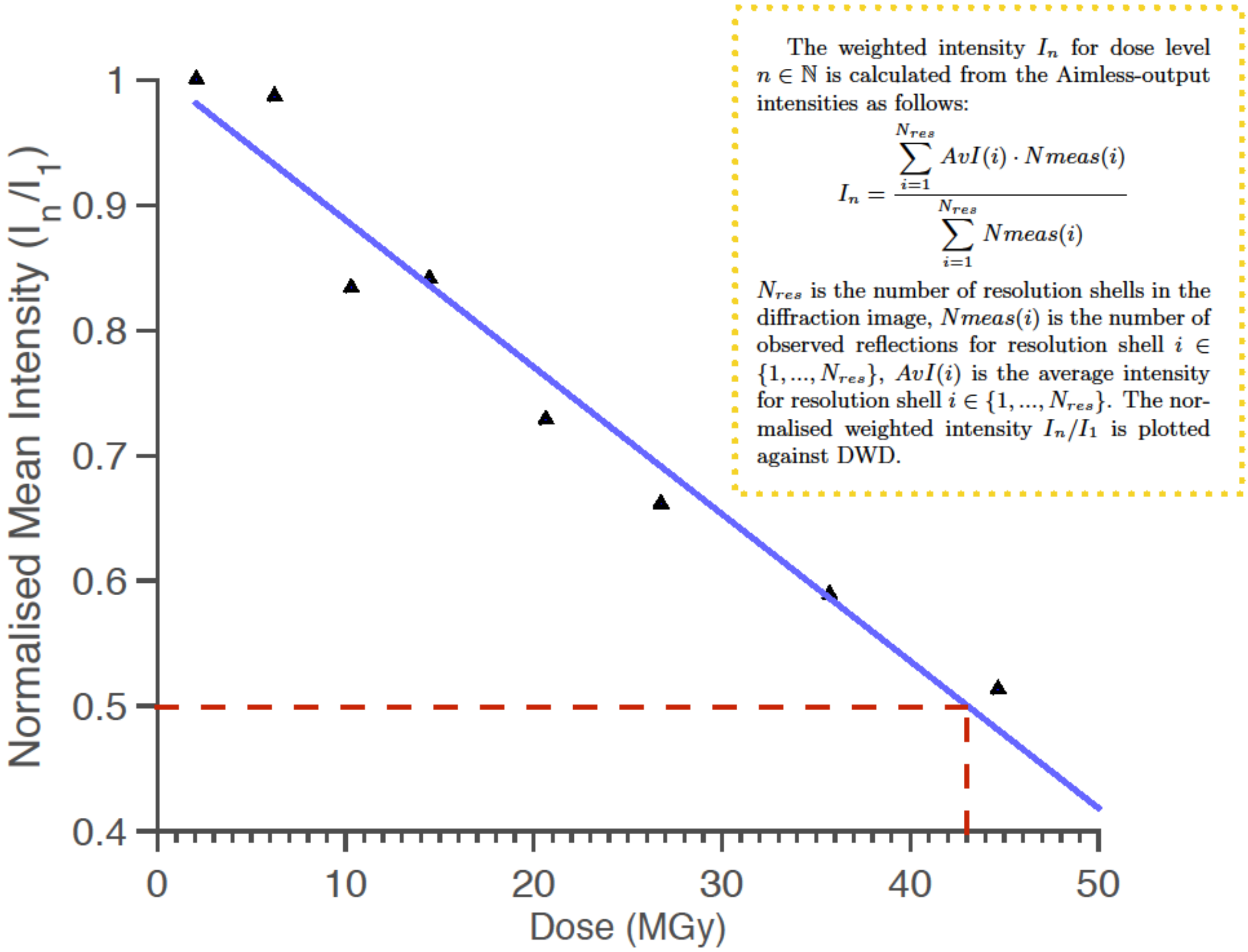

Figure S1 Mean intensity decay (69 Å - 2.8 Å) for the 8 C.Esp1396I datasets, normalised to the mean for the first dataset. The data are fitted with a straight line which gives a D1/2 of 43 MGy.

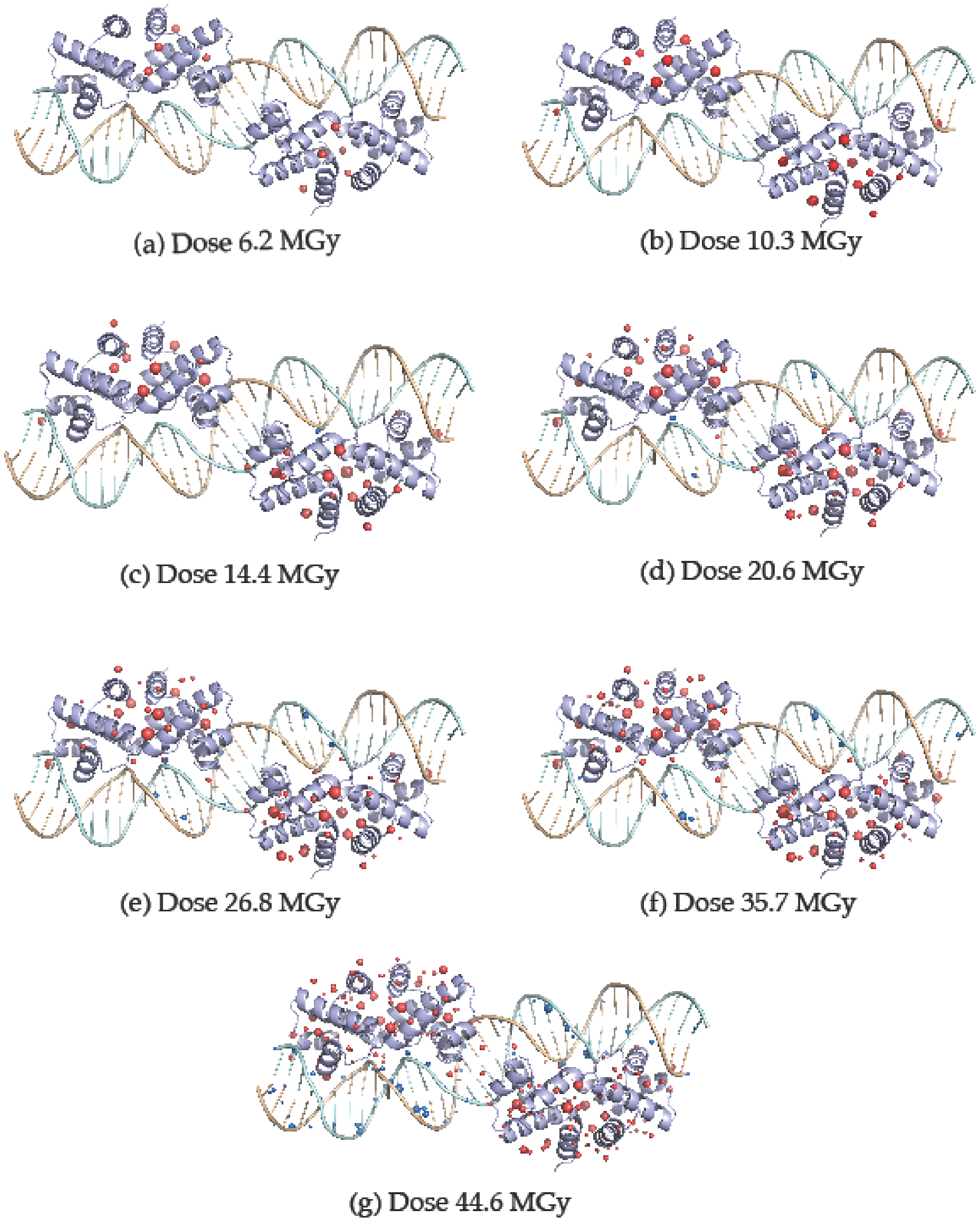

Figure S2 An extension of Figure 7 representing specific damage distribution throughout the C.Esp1396I complex for structures derived from all datasets across the full dose range 6.2 MGy to 44.6 MGy (a-g). Specific damage sites are represented as spheres, with van der Waals radii proportional to electron density loss (electrons per Å^3). Spheres closer/further than 2 Å to/from the DNA strands are coloured blue/red.
